# 

**Published:** 1904-04

**Authors:** 


### CONTENTS.

ORIGINAL COMMUNICATIONS—

THE IMPERFECT DEVELOPMENT OF THE UTERUS. By R. R. Kime, M.D., Atlanta, Ga. .......... 1
TOXIC DERMATOSES. By Bernard Wolff, M.D., Atlanta, Ga. .......... 6
CASES ILLUSTRATING PLASTIC SURGERY OF THE FACE. By M. B. Hutchins, M.D., Atlanta, Ga. .......... 12

SOCIETY REPORTS—

ATLANTA SOCIETY OF MEDICINE .......... 15
NORTHWESTERN BRANCH OF THE PHILADELPHIA COUNTY MEDICAL SOCIETY .......... 22

EDITORIAL NOTES AND COMMENTS—

PNEUMONIA .......... 33
ATLANTA COLLEGE OF PHYSICIANS AND SURGEONS .......... 35
GEORGIA SOCIOLOGICAL SOCIETY .......... 36
ON THE DEATH OF DR. VIRGIL O. HARDON .......... 37
RESOLUTIONS ADOPTED ON THE DEATH OF DR. VIRGIL O. HARDON .......... 38

NEWS AND NOTES .......... 39

CONTENTS CONTINUED .......... X

BOOK REVIEWS—

Diseases of the Eye. (Fox.) .......... 44

The Practical Medicine Series of Year Books. (Wood, Andrews, Head.) .......... 44

A System of Practical Surgery. (Van Bergmann, Von Bruns, Von Mikulicz) ... 45

Immune Sera. (Wassermann.) .......... 45

The Worth of Words. (Bell.) .......... 45

Progressive Medicine .......... 46

SELECTIONS AND ABSTRACTS—

The Treatment of Remittent and Intermittent (Malarial) Fevers .......... 47

Observations on Obstetrics in General Practice .......... 64

Catarrhal Diseases of the Upper Air-Passages .......... 69

Ambergris .......... 71

Cholelithiasis .......... 72

MEDICAL ITEMS—CONTINUED .......... xviii, xx, xxii, xxiv, xxvi, xxviii.

